# Structure of the Vitreous Humor

**Published:** 1849-08

**Authors:** 


### RECORD OF MEDICAL SCIENCE.

### ANATOMY AND PHYSIOLOGY.

*Structure of the Vitreous Humor.*—In a paper in the Dublin Quarterly Journal of Medical Science, for August, 1848, Mr. William Bowman, after noticing the researches of Pappenheim, of Brücke, and of Hannover, on the internal construction of the vitreous body, as disclosed by treating it for a certain period with solutions of carbonate of potass, of acetate of lead, and of chromic acid, and then making careful sections of it in various directions,—gives a precise and accurate account of his own examinations of this part in man, and in many of the inferior animals. From these researches we have extracted the following points.

In man, the vitreous humor, exposed to the action of dilute chromic acid for a year, and then sliced, displays a series of alternate light and dark layers, for the most part parallel to the outer surface. These extend inwards about one-eighth or one-fourth of an inch ; and in front seem to run up to the suspensory ligament of the lens, and to the posterior capsule. More internally, there was evidence of small canals traversing the vitreous body in a central direction ; while in its middle region there was an irregular cavity, apparently formed by breaking up of the tissue.

Mr. Bowman then adverts to the appearances produced in the vitreous body by bringing it into contact with solution of acetate of lead, according to the method of Brücke. In these experiments, the fresh eyes of mammals were used. The latter author placed the entire globe, or at least the entire vitreous body, in the solution ; and on finding a very beautiful system of concentric layers of precipitate, parallel to that surface, (formed after a certain period of immersion,) concluded that they arose from, and demonstrated the existence of, a true system of concentric membranes in the substance of the vitreous body,—the precipitate, which occasioned the white color, being, as he imagined, stopped in its progress inwards by the membranous sheets successively encountered.

Mr. Bowman, however, not feeling satisfied that his conclusion was a legitimate one, repeated and varied the experiments, by exposing to the action of the salt of lead, portions of the vitreous body previously cut in different directions,—and in all instances found the layers of precipitate deposited parallel to the surface, whether that surface were the hyaloid, or one formed by the knife. The necessary inference from this interesting fact, seems to be, that this elegant deposit of the precipitate, in distinct layers one within the other, is to be ascribed to some physical cause connected with the phenomenon of imbibition, and not to any pre-existing structure or arrangement of membranes. Of this cause no explanation can be afforded by the laws of endosmose, as at present known.

Mr. Bowman also describes the appearances produced in the vitreous body of a mature human fœtus, by the prolonged action of dilute chromic acid, and which are among the most interesting of the whole. He finds, at the region of the yellow spot, a cup-shaped evolution of the retina, into which the vitreous substance does not enter. The vitreous substance also exhibits a fibrous radiation from the hyaloid canal (that extending from the entrance of the optic nerve to the back of the lens, and conveying branches from the central artery and vein) towards the hyaloid surface, countenancing the opinion of Hannover, that the human vitreous body is formed in segments comparable to those of an orange. In the coagulated vitreous of the human fœtus, Mr. Bowman finds an elegant fibrous structure, like that of the enamel pulp.

The author attributes no value to the assumed evidences of structure afforded by congelation of the vitreous body. Mr. Bowman then explains the results of his observations on the vitreous body, in birds and fishes; and concludes that, in the latter, especially, there is good ground for believing that it contains a true system of lamellæ, proceeding from the junction of the choroid and iris, to the lens, and contributing to the mechanical support of this large and solid part in the eye of the fish.—*London Journal of Medicine.*

